# Dimension reduction with redundant gene elimination for tumor classification

**DOI:** 10.1186/1471-2105-9-S6-S8

**Published:** 2008-05-28

**Authors:** Xue-Qiang Zeng, Guo-Zheng Li, Jack Y Yang, Mary Qu Yang, Geng-Feng Wu

**Affiliations:** 1 School of Computer Engineering & Science, Shanghai University, Shanghai 200072, China; 2 State Key Laboratory for Novel Software Technology, Nanjing University, Nanjing 210093, China; 3 Harvard Medical School, Harvard University, Cambridge, Massachusetts 02140 USA; 4 National Human Genome Research Institute National Institutes of Health (NIH) U.S., Department of Health and Human Services Bethesda, MD 20852 USA

## Abstract

**Background:**

Analysis of gene expression data for tumor classification is an important application of bioinformatics methods. But it is hard to analyse gene expression data from DNA microarray experiments by commonly used classifiers, because there are only a few observations but with thousands of measured genes in the data set. Dimension reduction is often used to handle such a high dimensional problem, but it is obscured by the existence of amounts of redundant features in the microarray data set.

**Results:**

Dimension reduction is performed by combing feature extraction with redundant gene elimination for tumor classification. A novel metric of redundancy based on DIScriminative Contribution (DISC) is proposed which estimates the feature similarity by explicitly building a linear classifier on each gene. Compared with the standard linear correlation metric, DISC takes the label information into account and directly estimates the redundancy of the discriminative ability of two given features. Based on the DISC metric, a novel algorithm named REDISC (Redundancy Elimination based on Discriminative Contribution) is proposed, which eliminates redundant genes before feature extraction and promotes performance of dimension reduction. Experimental results on two microarray data sets show that the REDISC algorithm is effective and reliable to improve generalization performance of dimension reduction and hence the used classifier.

**Conclusion:**

Dimension reduction by performing redundant gene elimination before feature extraction is better than that with only feature extraction for tumor classification, and redundant gene elimination in a supervised way is superior to the commonly used unsupervised method like linear correlation coefficients.

## Background

DNA microarray experiments are used to collect information from tissue and cell samples regarding gene expression differences for tumor diagnosis [1,2]. The output of microarray experiment is summarized as an *n *× *p *data matrix, where *n *is the number of tissue or cell samples, *p *is the number of genes (features). Here, *p *is always much larger than *n*, which hurts generalization performance of most classification methods. To overcome this problem, we either select a small subset of interesting genes (gene selection, feature selection) or construct *K *new components summarizing the original data as well as possible, with *K *<*p *(feature extraction).

Gene selection has been studied extensively in the last few years. The most commonly used procedures of gene selection are based on a score which is calculated for all genes individually and genes with the best scores are selected. Gene selection procedures output a list of relevant genes which can be experimentally analyzed by biologists. The method is often denoted as univariate gene selection, whose advantages are its simplicity and interpretability. However, interactions and correlations between genes are omitted during gene selection, although they are of great interest in system biology. Furthermore, gene selection often fails to pick relevant genes, because the score they assign to correlated genes is too similar, and none of the genes is strongly preferred over another.

Feature extraction is an alternative to gene selection to overcome curse of dimensionality. Unlike gene selection, feature extraction projects the whole data into a low dimensional space and constructs new dimensions (components) by analyzing the statistical relationship hidden in the data set. Although feature extraction is often criticized for the lack of interpretability, the new components often give good information or hints about the data's intrinsic structure. Researchers have developed different feature extraction methods in applications of bioinformatics and computational biology [3-5], which are generally divided into two groups, unsupervised and supervised. Among various methods, Principle Component Analysis (PCA), an unsupervised method, and Partial Least Squares (PLS), a supervised method, are widely used [5].

Considering of the fact that gene selection and feature extraction algorithms have complementary advantages and disadvantages. Feature extraction algorithms thrive on correlation among features but fail to remove irrelevant and redundant features from a set of complex features. Feature selection algorithms fail when all the features are correlated but do well with informative features. It would be an interesting work to combine gene selection and feature extraction into a general model. In practical, the simplest way is to apply a preliminarily gene selection procedure before feature extraction.

As to analysis of microarray data whose speciality is the huge amount of genes with few examples, it is believed that there exist many redundant genes among the full gene set [6]. Preserving the most discriminative genes and reducing other irrelevant and redundant genes still remain as an open issue. In this paper, we propose a novel metric of redundancy which can effectively eliminate redundant genes before feature extraction. By measuring the discriminative ability of each gene and the pair-wise complementarity, the new method reduce the redundant genes with little contribution of discriminative ability. We also compare our method with commonly used redundant gene reduction methods based on linear correlation. Experiments on several real microarray data sets demonstrate the outstanding performance of our method.

Some notions used in this work are clarified here. Expression levels of *p *genes in *n *microarray samples are collected in an *n *× *p *data matrix *X *= (*x*_*ij*_), 1 ≤ *i *≤ *n*, 1 ≤ *j *≤ *p*; of which an entry *x*_*ij *_is the expression level of the *j*th gene in the *i*th microarray sample. As we only consider binary classification problems, the labels of the *n *microarray samples are collected in the vector **y**. When the *i*th sample belongs to class one, the element *y*_*i *_is 1; otherwise it is -1. The matrix *S*_*X *_denotes the *p *× *p *covariance matrix of the gene expressions.

Besides, || • || denotes the length of a vector. *X*^*T *^represents the transpose of *X*, *X*^-1 ^represents the inverse matrix of *X*. The matrices *X *and **y **used in the following are assumed to be centered to zero mean by each column.

## Results and discussion

### Results

According to the framework proposed in this paper, dimension reduction is performed by combining redundant gene elimination with feature extraction, then the classifier is used to perform classification on the extracted feature subsets. The novel proposed algorithm REDISC (Redundancy Elimination based on DIScriminative Contribution) is compared with the commonly used algorithm RELIC (Redundancy Elimination based on LInear Correlation) to perform redundant gene elimination on two microarray data sets, i.e. Colon and Leukemia, where the threshold of δ in REDISC and RELIC is varied from 0.1 to 0.9. Feature extraction is performed by principle component analysis (PCA) and partial least squares (PLS). The classifier is a linear support vector machine (SVM) with *C *= 1.

**Figure 1:**
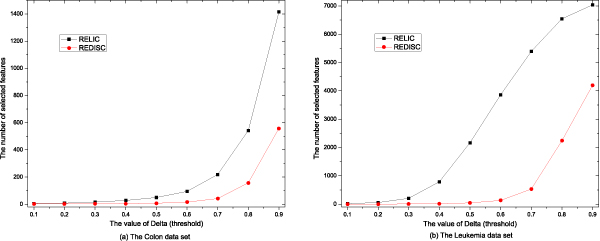
The number of selected genes by performing REDISC and RELIC with different parameters.

Statistical results of the number of remained genes after performing REDISC and RELIC are showed in Figure 1, the detailed results are also listed in Tables 1-4.

**Table 1:** Statistical results by performing PLS after REDISC and RELIC with different parameters on the Colon data set

	#genes	Sensitivity	Specificity	BACC	Precision	PPV	NPV	Correction
RELIC 0.1	5.62	0.9750	0.3167	0.6458	0.7362	0.7362	0.5217	0.7424
RELIC 0.2	8.56	0.9600	0.4167	0.6883	0.7646	0.7646	0.6400	0.7667
RELIC 0.3	16.55	0.9350	0.5567	0.7458	0.8131	0.8131	0.7258	0.7995
RELIC 0.4	28.08	0.9150	0.6650	0.7900	0.8468	0.8468	0.8208	0.8264
RELIC 0.5	49.55	0.9100	0.7017	0.8058	0.8633	0.8633	0.8092	0.8352
RELIC 0.6	94.3	0.8975	0.7133	0.8054	0.8682	0.8682	0.7992	0.8329
RELIC 0.7	218.37	0.8950	0.7650	0.8300	0.8955	0.8955	0.8075	0.8490
RELIC 0.8	542.46	0.8825	0.7917	0.8371	0.9003	0.9003	0.8187	0.8500
RELIC 0.9	1413.92	0.8750	0.7967	0.8358	0.9080	0.9080	0.8110	0.8479

REDISC 0.1	2	1.0000	0.1850	0.5925	0.6996	0.6996	0.3600	0.7121
REDISC 0.2	2.1	0.9850	0.2083	0.5967	0.7022	0.7022	0.3950	0.7107
REDISC 0.3	2.94	0.9800	0.2983	0.6392	0.7315	0.7315	0.4883	0.7383
REDISC 0.4	4.3	0.9750	0.3750	0.6750	0.7549	0.7549	0.6050	0.7617
REDISC 0.5	8.09	0.9200	0.5133	0.7167	0.7911	0.7911	0.7133	0.7752
REDISC 0.6	15.82	0.8975	0.6533	0.7754	0.8443	0.8443	0.7525	0.8112
REDISC 0.7	41.9	0.9000	0.7800	0.8400	0.8992	0.8992	0.8208	0.8579
REDISC 0.8	157.06	0.8950	0.8150	0.8550	0.9150	0.9150	0.8350	0.8662
REDISC 0.9	558	0.8900	0.7900	0.8400	0.8985	0.8985	0.8277	0.8533

Full Set	2000	0.8750	0.7733	0.8242	0.8958	0.8958	0.8137	0.8388

**Table 2:** Statistical results by performing PCA after REDISC and RELIC with different parameters on the Colon data set

	#genes	Sensitivity	Specificity	BACC	Precision	PPV	NPV	Correction
RELIC 0.1	5.62	0.9750	0.2817	0.6283	0.7231	0.7231	0.4800	0.7298
RELIC 0.2	8.56	0.9625	0.3917	0.6771	0.7546	0.7546	0.6333	0.7605
RELIC 0.3	16.55	0.9300	0.5483	0.7392	0.8075	0.8075	0.7267	0.7931
RELIC 0.4	28.08	0.9175	0.6650	0.7912	0.8480	0.8480	0.8142	0.8279
RELIC 0.5	49.55	0.9075	0.7033	0.8054	0.8645	0.8645	0.8075	0.8338
RELIC 0.6	94.3	0.9125	0.7250	0.8188	0.8743	0.8743	0.8208	0.8455
RELIC 0.7	218.37	0.9025	0.7917	0.8471	0.9053	0.9053	0.8483	0.8631
RELIC 0.8	542.46	0.8725	0.7883	0.8304	0.8987	0.8987	0.8102	0.8424
RELIC 0.9	1413.92	0.9000	0.7400	0.8200	0.8802	0.8802	0.8175	0.8426

REDISC 0.1	2	0.9975	0.0150	0.5063	0.6516	0.6516	0.0200	0.6510
REDISC 0.2	2.1	0.9875	0.0550	0.5213	0.6599	0.6599	0.1000	0.6590
REDISC 0.3	2.94	0.9800	0.2167	0.5983	0.7039	0.7039	0.3950	0.7095
REDISC 0.4	4.3	0.9675	0.3350	0.6513	0.7396	0.7396	0.5483	0.7424
REDISC 0.5	8.09	0.9175	0.5017	0.7096	0.7875	0.7875	0.6833	0.7690
REDISC 0.6	15.82	0.8975	0.6533	0.7754	0.8443	0.8443	0.7525	0.8112
REDISC 0.7	41.9	0.9000	0.7750	0.8375	0.8978	0.8978	0.8108	0.8562
REDISC 0.8	157.06	0.8900	0.8117	0.8508	0.9142	0.9142	0.8300	0.8617
REDISC 0.9	558	0.8750	0.7483	0.8117	0.8828	0.8828	0.7892	0.8298

Full Set	2000	0.8925	0.7150	0.8038	0.8680	0.8680	0.7950	0.8290

**Table 3:** Statistical results by performing PLS after REDISC and RELIC with different parameters on the Leukemia data set

	#genes	Sensitivity	Specificity	BACC	Precision	PPV	NPV	Correction
RELIC 0.1	18.1	0.9400	0.7567	0.8483	0.8872	0.8872	0.8898	0.8743
RELIC 0.2	55.86	0.9110	0.7850	0.8480	0.9024	0.9024	0.8318	0.8663
RELIC 0.3	205.48	0.9415	0.8150	0.8783	0.9161	0.9161	0.8842	0.8964
RELIC 0.4	790.41	0.9605	0.8133	0.8869	0.9141	0.9141	0.9200	0.9084
RELIC 0.5	2168.33	0.9720	0.9017	0.9368	0.9537	0.9537	0.9575	0.9470
RELIC 0.6	3859.52	0.9795	0.9550	0.9672	0.9782	0.9782	0.9742	0.9711
RELIC 0.7	5394.2	0.9795	0.9500	0.9647	0.9752	0.9752	0.9742	0.9686
RELIC 0.8	6545.28	0.9815	0.9150	0.9483	0.9585	0.9585	0.9750	0.9573
RELIC 0.9	7035.99	0.9840	0.9067	0.9453	0.9572	0.9572	0.9775	0.9571

REDISC 0.1	3.31	0.9865	0.6317	0.8091	0.8394	0.8394	0.9267	0.8595
REDISC 0.2	4.29	0.9805	0.6683	0.8244	0.8566	0.8566	0.9050	0.8700
REDISC 0.3	6.88	0.9695	0.7833	0.8764	0.9052	0.9052	0.9208	0.9041
REDISC 0.4	14.83	0.9635	0.8783	0.9209	0.9418	0.9418	0.9475	0.9316
REDISC 0.5	46.56	0.9710	0.9533	0.9622	0.9760	0.9760	0.9625	0.9641
REDISC 0.6	131.23	0.9665	0.9633	0.9649	0.9810	0.9810	0.9567	0.9654
REDISC 0.7	531.33	0.9775	0.9450	0.9612	0.9723	0.9723	0.9700	0.9657
REDISC 0.8	2239.81	0.9855	0.9383	0.9619	0.9702	0.9702	0.9842	0.9686
REDISC 0.9	4195.03	0.9885	0.9117	0.9501	0.9600	0.9600	0.9858	0.9613

Full Set	7129	0.9840	0.9083	0.9462	0.9572	0.9572	0.9775	0.9573

**Table 4:** Statistical results by performing PCA after REDISC and RELIC with different parameters on the Leukemia data set

	#genes	Sensitivity	Specificity	BACC	Precision	PPV	NPV	Correction
RELIC 0.1	18.1	0.9395	0.7433	0.8414	0.8818	0.8818	0.8758	0.8702
RELIC 0.2	55.86	0.9115	0.7783	0.8449	0.9024	0.9024	0.8007	0.8646
RELIC 0.3	205.48	0.9440	0.7700	0.8570	0.8960	0.8960	0.8692	0.8827
RELIC 0.4	790.41	0.9585	0.7983	0.8784	0.9105	0.9105	0.8975	0.9027
RELIC 0.5	2168.33	0.9735	0.8783	0.9259	0.9441	0.9441	0.9617	0.9405
RELIC 0.6	3859.52	0.9865	0.9400	0.9632	0.9716	0.9716	0.9825	0.9700
RELIC 0.7	5394.2	0.9910	0.9567	0.9738	0.9786	0.9786	0.9883	0.9784
RELIC 0.8	6545.28	0.9910	0.9633	0.9772	0.9815	0.9815	0.9875	0.9807
RELIC 0.9	7035.99	0.9955	0.9633	0.9794	0.9815	0.9815	0.9933	0.9836

REDISC 0.1	3.31	0.9865	0.6267	0.8066	0.8380	0.8380	0.9167	0.8579
REDISC 0.2	4.29	0.9805	0.6583	0.8194	0.8550	0.8550	0.8858	0.8671
REDISC 0.3	6.88	0.9695	0.7733	0.8714	0.9013	0.9013	0.9108	0.9000
REDISC 0.4	14.83	0.9655	0.8700	0.9178	0.9385	0.9385	0.9508	0.9304
REDISC 0.5	46.56	0.9735	0.9417	0.9576	0.9715	0.9715	0.9642	0.9614
REDISC 0.6	131.23	0.9690	0.9467	0.9578	0.9737	0.9737	0.9592	0.9611
REDISC 0.7	531.33	0.9795	0.9517	0.9656	0.9765	0.9765	0.9742	0.9698
REDISC 0.8	2239.81	0.9880	0.9600	0.9740	0.9798	0.9798	0.9867	0.9782
REDISC 0.9	4195.03	0.9980	0.9550	0.9765	0.9787	0.9787	0.9967	0.9823

Full Set	7129	0.9955	0.9633	0.9794	0.9815	0.9815	0.9933	0.9836

**Figure 2:**
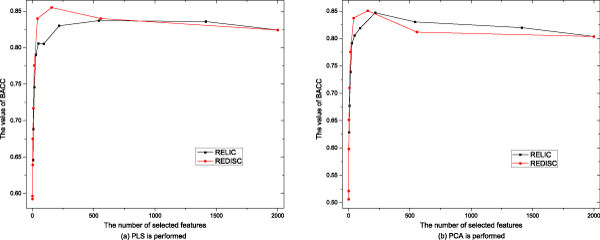
Comparative results of BACC scores by using different algorithms on the Colon data set.

**Figure 3:**
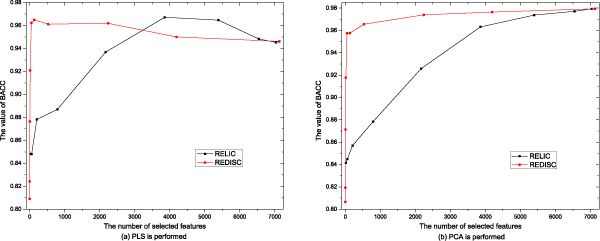
Comparative results of BACC scores by using different algorithms on the Leukemia data set.

Comparative results of BACC obtained by SVM on the new feature sets by using PCA or PLS after performing REDISC and RELIC are illustrated in Figure 2 and Figure 3. Detailed results of Sensitivity, Specificity, BACC, Precision, PPV, NPV and correction on Colon and Leukemia are showed in Tables 1-4, where the results are averaged on ten times of run.

The results in Figures 1-3 and Tables 1-4 show that:

1. Both REDISC and RELIC dramatically reduce the number of genes from the original data. With the same value of *δ*, REDISC obtains more compact subsets than RELIC does.

2. With *δ *= 0:1, RELIC always obtains better results than REDISC, but when δ increases, the results of REDISC are better than those of RELIC. On average, REDISC obtains better results than RELIC does.

3. When the results of REDISC and RELIC reach their highest point, REDISC uses less features than RELIC.

4. The effect of REDISC is positive for both PCA and PLS, while RELIC loses in some case, i.e. BACC of PCA on the Leukemia data set.

5. REDISC and RELIC with different threshhold values produces different results, no one is optimal for all the data sets.

### Discussion

The experimental results prove our assumption that redundant features hurt performance of feature extraction and classification, other considerations on the above results are listed as below:

1. The results confirm that there exist many redundant genes in the microarray data and it is necessary to perform redundant gene elimination. Usually, there are four types of features in one data set, I is strong relevant features, II is weak relevant but non redundant features, III is weak relevant and redundant features and IV is irrelevant features. I and II are the essential features in the data sets, and III and IV should be removed [7]. The previous works show III and IV should be removed for classifiers, and in this paper, we show they should also be removed for feature extraction like PCA and PLS.

2. REDISC obtains better results with less features than RELIC, which shows that REDISC has the higher ability to select relevant features and eliminate the redundant features than RELIC. Proper redundant feature elimination help improve performance of feature extraction and classification. Simply reducing redundant genes by linear correlation is not always positive, because without considering the label information in the data set, linear correlation does not give properly redundancy estimation. REDISC takes label information into account for redundant gene elimination, which may be viewed as a supervised way. Since the final step is classification, so a supervised redundant gene elimination is better than an unsupervised one like RELIC.

3. It shows the performances of dimension reduction is improved when redundant genes are properly eliminated. The improvement for PLS is much more dramatic than that of PCA. A possible reason is redundant genes obstruct the performance of supervised methods more obviously, since supervised methods often build more precisely model than unsupervised ones.

## Conclusion

Dimension Reduction is widely used in bioinformatics and related fields to overcome the curse of dimensionality. But the existence of amounts of redundant genes in the microarray data often obscure the application of dimension reduction. Preliminarily redundant gene elimination before feature extraction for dimension reduction is an interesting issue, which was often neglected.

In this paper, a novel metric of redundancy based on Discriminative Contribution (DISC) is proposed, which directly estimates the similarity between two features by explicitly building linear classifiers on each genes. The REDISC algorithm (Redundancy Elimination based on Discriminative Contribution) is also proposed. REDISC is compared with a commonly used algorithm RELIC (Redundancy Elimination based on Linear Correlation) on two real microarray data sets. Experimental results demonstrate the necessariness of preliminarily redundant gene elimination before feature extraction for tumor classification and the superiority of REDISC to RELIC, a commonly used method. This work is an attempt to propose a general framework performing dimension reduction for tumor classification by combing redundant gene elimination and feature extraction. More investigation need to be done on the efficiency of fusion of feature selection with feature extraction in the future.

## Methods

### A framework of dimension reduction

**Figure 4:**
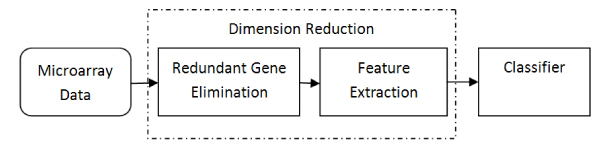
The novel framework of dimension reduction.

In this paper, we propose a novel framework for dimension reduction by combining redundant feature elimination with feature extraction to improve performance of classification. The framework is illustrated as in Figure 4, where the microarray data is performed dimension reduction before classification, and dimension reduction consists of redundant gene elimination and feature extraction. The algorithms of redundant gene elimination before feature extraction in this paper actually remove irrelevant features and redundant features at the same time. We omit irrelevant gene elimination because irrelevant genes are few in the gene data set and are not the focus in this paper.

Redundant gene elimination is the critical part in the framework, we propose a novel algorithm based on discriminative ability to improve performance of commonly used linear correlation, which is described in detail in the following subsections. Feature extraction is performed by using two methods, one is supervised, i.e. partial least squares, another is unsupervised, i.e. principle component analysis, which are briefly introduced in the following subsection. As for the classifier, support vector machine is used.

### Redundant gene elimination

As redundant features have no contribution for classification, we consider eliminating them preliminarily before feature extraction, which has the following benefits:

1. Eliminating redundant features improves classification accuracy. In general, original microarray data sets have many irrelevant and redundant genes, which hurts performance of feature extraction. In practical, biologists often expect noises are reduced, at least in some extent, during the stage of feature extraction. But, if some redundant genes are reduced beforehand, performance of feature extraction may be improved.

2. Preliminarily feature selection facilitates the application of feature extraction. Compared with modeling on the original data directly, the computational and RAM consumptions of feature extraction on preliminarily gene selected data are much less. Especially for the RAM consumption, most feature extraction methods are often not practical for high dimensional data, since the requirement of loading all data into RAM at one time. However, any additional gene selection procedure may bring some extra computation, so the computational complexity of preliminarily feature selection must not be too high.

3. Preliminarily feature selection improves the interpretability of the components. The meanings of the components are always difficult to be interpreted in feature extraction. Biologists often analyze the relation between extracted components and original features by the coefficients, but it is obscured by the large amount of genes. Reducing a number of original features is obviously helpful when the components are needed to be related with original genes manually.

#### The previous metrics

Discriminative ability (predictive ability) is a general notion which can be measured in various ways and be used to select significant features for classification. Many effective metrics had been proposed such as t-statistic, information gain, *χ*^2 ^statistic, odds ratio *etc*. [8,9]. Filter feature selection methods sort features by the discriminative ability scores, and some top rank features are retained to be essential for classification.

However, t-statistic and most of other discriminative ability measures are based on individual features, which do not consider the redundancy between two features. Because given two features with the same rank scores, they may be redundant to each other when they are completely correlated, otherwise, they may also be complementary to each other when they are nearly independent.

For the task of feature selection, we want to eliminate the redundant features and only retain the interactive ones. But there exist many redundant features in the top rank feature set produced by using the filter methods. The redundant features increase the dimensionality and contribute little for the final classification. In order to eliminate redundant features, metrics need to estimate the redundancy directly.

Notions of feature redundancy are normally in terms of feature correlation. It is widely accepted that two features are redundant to each other if their values are completely correlated. But in fact, it may not be so straightforward to determine feature redundancy when a feature is correlated with a set of features. The widely used way is to approximate the redundancy of feature set by considering the pair-wise feature redundancy.

##### The linear correlation metric

For linear cases, the most well known pair-wise redundancy metric is the linear correlation coefficient. Given a pair of features (*x*, *y*), the definition of the linear correlation coefficient Cor(*x*, *y*) is:

(1) $$\text{Cor}(x,y)=\frac{\Sigma_{i}(x_{i}-\bar{x})(y_{i}-\bar{y})}{\sqrt{\Sigma_{i}{\left(x_{i}-\bar{x}\right)}^{2}}\sqrt{\Sigma_{i}{\left(y_{i}-\bar{y}\right)}^{2}}}$$

where $\bar{x}$ and $\bar{y}$ are the mean of *x *and *y *respectively. The value of Cor(*x*, *y*) lies between -1 and 1. If *x *and *y *are completely correlated, Cor(*x*, *y*) takes the value of 1 or -1; if *x *and *y *are independent, Cor(*x*, *y*) is zero. It is a symmetrical metric.

The linear correlation coefficient has the advantage of its efficiency and simplicity, but it is not suitable for redundant feature elimination when classification is the final target, since it does not use any label information. For example, two highly correlated features, whose differences are minor in values but happen to causing different critical discriminative ability, may be considered as a pair of redundancy features. Reducing any one of them will decrease classification accuracy. Guyon et al. has also pointed out that high correlation (or anti-correlation) of variables does not mean absence of variable complementarity [8]. The problem of the linear correlation coefficient is that it measures the similarity of the numerical values between two features, but not the similarity of discriminative ability between two features.

The ideal feature set should have both great discriminative ability and little feature redundancy, where redundancy could not be obtained by estimating their properties separately. A more elaborate measure of redundancy is required to estimate the differences of the discriminative ability between two features.

#### The proposed novel metric

In order to measure the similarity of discriminative ability of two features, the discriminative ability need be defined more precisely. That is to say, we want to know which example can be rightly classified by the given feature and which can not. Upon the new metric, it is possible to compare the discriminative ability of two features by the corresponding correctly classified examples.

In the field of text classification, Training Accuracy on Single Feature (TASF) has been proved to be an effective metric of discriminative ability [9], which builds a classifier for each feature, and the corresponding training accuracy is used as the discriminative score.

Various classifiers can be used to calculate TASF, in simplification, we consider a linear learner here. Given a feature *z*, the classification function is given as:

(2) $$\hat{y}=\mathrm{sgn}(({\bar{z}}^{1}-{\bar{z}}^{2})(z-\frac{n^{1}{\bar{z}}^{1}+n^{2}{\bar{z}}^{2}}{n^{1}+n^{2}}))$$

where ${\bar{z}}^{1}$ and *n*^1 ^are the feature mean and the sample size of class one, ${\bar{z}}^{2}$ and *n*^2 ^are the feature mean and the sample size of class two. This is a weighted centroid based classifier, which predicts examples as the class label whose weighted distance to its centroid is smaller. The computational complexity of this classifier is *O*(*n*).

Putting the whole training set back, we can estimate training accuracy of each classifier by different features, which is used to represent discriminative ability of the corresponding feature. The higher training accuracy, the greater discriminative ability. Since only one feature is used to build the classifier, a part of training examples can be correctly separated in most cases. So the value of TASF ranges from 0 to 1. One feature is considered as an irrelevant one if its TASF value is no greater than 0.5.

Based on TASF, we propose a novel metric of feature redundancy. Given two features of *z*_1 _and *z*_2_, two classifiers *C*_1 _and *C*_2 _can be constructed. Feeding the whole training set to the classifiers, both *C*_1 _and *C*_2 _can correctly classify a sample subset. The differences of the correctly classified examples are used to estimate the similarity of discriminative abilities. We record the concrete classification results as in table 5, where *a *+ *b *+ *c *+ *d *equals to the size of the training set *n*. The values of (*a *+ *b*)/*n *and (*a *+ *c*)/*n *are training accuracy of *C*_1 _and *C*_2 _respectively. The score of *a *+ *d *measures the similarity of the features, and the score of *b *+ *c *measures the dissimilarity. When *b *+ *c *= 0, the two features *z*_1 _and *z*_2 _have exactly the same discriminative ability.

**Table 5:** Statistical relative classification results of two classifiers

*C*_1_/*C*_2_	true	false
true	a	b
false	c	d

Our feature elimination problem is becoming whether the contribution of the additional feature to the given feature is significant. The additional feature is considered as redundant if its contribution is tiny. Then, we propose a novel metric of Redundancy based on DIScriminative Contribution (DISC). DISC of *z*_1 _and *z*_2_, which estimates *z*_2_'s redundancy to *z*_1_, is defined as follows,

(3) $$\begin{array}{c} \text{DISC}(z_{1},z_{2})=1-\frac{c}{c+d}\\ =\frac{d}{c+d} \end{array}$$

The pair-wise DISC metric is asymmetrical, and the computation complexity is *O*(*n*).

It is clear that *c *+ *d *is the number of examples which could not be discriminated by *C*_1_, *c *is that which could be correctly classified by the collaboration of *C*_1 _and *C*_2_. So the proportion of *c*/(*c *+ *d*) is the discriminative contribution of *C*_2 _to *C*_1_, and the value of *d*/(*c *+ *d*) is the DISC metric of redundancy, which varies from 0 to 1. When the DISC score takes 1, *C*_2_'s discriminative ability is covered by *C*_1_'s and then *z*_2 _is completely redundant to *z*_1_. When the DISC value is 0, all training examples could be correctly classified by the union of *C*_2 _and *C*_1 _and we consider *z*_2 _is complementary to *z*_1_.

DISC is proposed in a linear way, which shows in two respects, one is the linear classifier, another is the linear way of counting the cross discriminative abilities. The microarray problems meet the assumption, since most microarray data sets are binary classification problems, where each gene has equal position to perform classification.

#### The proposed redundant gene elimination algorithms

##### The REDISC algorithm

**Figure 5:**
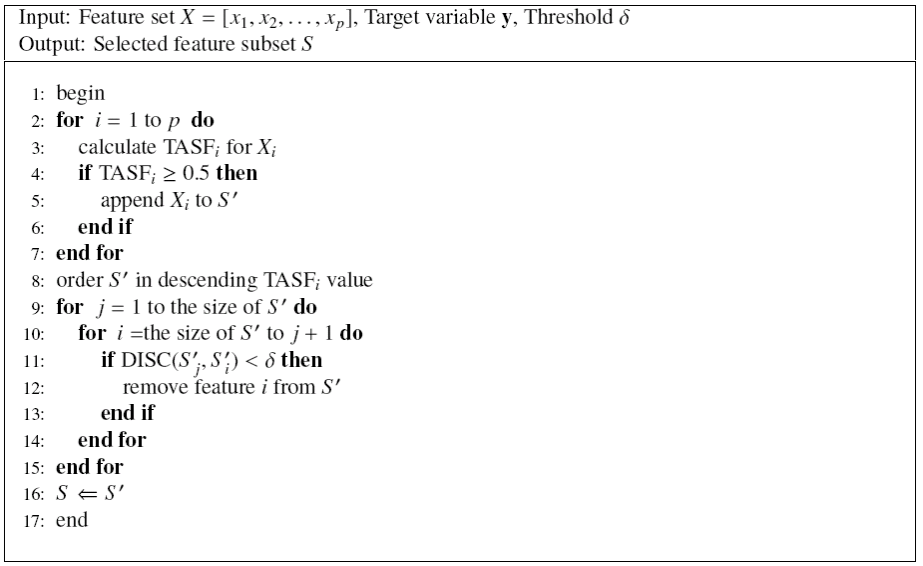
The REDISC algorithm.

Based on the DISC redundancy metric, we propose the REDISC algorithm (Redundancy Elimination based on Discriminative Contribution), which eliminates redundant features by the pair-wise DISC scores. REDISC is illustrated in Figure 5, its basic idea is that, firstly, REDISC filters out trivial features, which do not have discriminative ability on itself, by the TASF score threshold of 0.5. Then the features are ordered by their TASF scores. As we usually want to retain the more discriminative one between two redundant features, REDISC tries to preserve the top TASF score ranked features. REDISC uses two nested iterations to eliminate redundant features whose discriminative ability are covered by any higher ranked features. The computational complexity of REDISC is *O*(*np*^2^).

##### The RELIC algorithm

**Figure 6:**
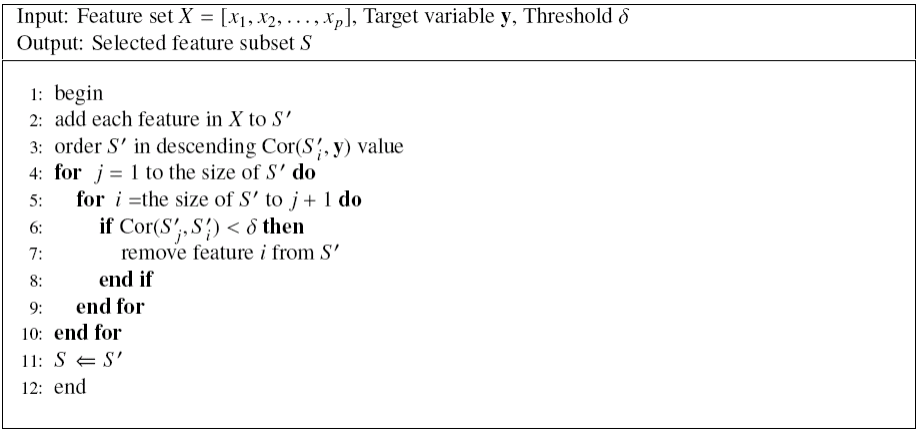
The RELIC algorithm.

In order to compare our method with commonly used redundant feature elimination methods, we present the algorithm of RELIC (Redundancy Elimination based on Linear Correlation) [10], which filters out redundant features by the pair-wise linear correlation. A threshold is needed to control how many features should be eliminated. RELIC is given in Figure 6, whose computational complexity is also *O*(*np*^2^).

### Feature extraction techniques

#### Principle component analysis

Principle component analysis (PCA) is a well-known method of feature extraction [11]. The basic idea of PCA is to reduce the dimensionality of a data set, while retaining as much as possible the variation in the original predictor variables. This is achieved by transforming the *p *original variables *X *= [*x*_1_, *x*_2_, ..., *x*_*p*_] to a new set of *K *predictor variables, *T *= [*t*_1_, *t*_2_, ..., *t*_*K*_], which are linear combinations of the original variables. In mathematical terms, PCA sequentially maximizes the variance of a linear combination of the original predictor variables,

(4) $$u_{K}=\underset{u^{T}u=1}{\arg\max}(\text{Var}(Xu))$$

subject to the constraint $u_{i}^{T}S_{X}u_{j}=0$, ∀ 1 ≤ *i *<*j*. The orthogonal constraint ensures that the linear combinations are uncorrelated, *i.e*. Cov(*X***u**_*i*_, *X***u**_*j*_) = 0, *i *≠ *j*. These linear combinations

(5) *t*_*i *_= *X***u**_*i*_

are known as the principal components (PCs).

The maximum number of components *K *is determined by the number of nonzero eigenvalues, which is the rank of *S*_*X*_, and *K *≤ min(*n*, *p*). But in practical, the maximum value of *K *is not necessary. Some tail components, which have tiny eigenvalues and represent few variances of original data, are often needed to be reduced. The threshold of *K *often determined by cross-validation or the proportion of explained variances [11]. The computational cost of PCA, determined by the number of original predictor variables *p *and the number of samples *n*, is in the order of min(*np*^2 ^+ *p*^3^, *pn*^2 ^+ *n*^3^). In other words, the cost is *O*(*pn*^2 ^+ *n*^3^) when *p *> *n*.

#### Partial Least Squares

Partial Least Squares (PLS) was firstly developed as an algorithm performing matrix decompositions, and then was introduced as a multivariate regression tool in the context of chemometrics [12,13]. In recent years, PLS has also been found to be an effective feature extraction technique for tumor discrimination [14,15].

The underlying assumption of PLS is that the observed data is generated by a system or process which is driven by a small number of latent (not directly observed or measured) features. Therefore, PLS aims at finding uncorrelated linear transformations (latent components) of the original predictor features which have high covariance with the response features. Based on these latent components, PLS predicts response features **y**, the task of regression, and reconstruct original matrix *X*, the task of data modeling, at the same time.

The objective of constructing components in PLS is to maximize the covariance between the response variable **y **and the original predictor variables *X*,

(6) $$w_{K}=\underset{w^{T}w=1}{\arg\max}(\text{Cov}(Xw,y))$$

subject to the constraint $w_{i}^{T}S_{X}w_{j}=0$, ∀ 1 ≤ *i *<*j*. The central task of PLS is to obtain the vectors of optimal weights **w**_*i *_(*i *= 1, ..., *K*) to form a small number of components, while PCA is an "unsupervised" method that utilizes the *X *data only.

Like PCA, PLS reduces the complexity of microarray data analysis by constructing a small number of gene components, which can be used to replace the large number of original gene expression measures. Moreover, obtained by maximizing the covariance between the components and the response variable, the PLS components are generally more predictive of the response variable than the principal components.

PLS computes efficiently with a cost only at *O*(*npK*), *i.e*. the number of calculations required by PLS is a linear function in terms of *n *or *p*. Thus it is much faster than the method of PCA for *K *is always less than *n*.

Feature extraction methods extract components to represent original data, which are linear or non-linear transformations of original genes. Although the new subspace is effective for data analysis, no original gene is excluded during the process, which often obstructs the explanations of PCs. In order to solve this problem, eliminating redundant genes before dimension reduction is an alternative way.

### Classifier – Support Vector Machines

Support vector machines (SVM) proposed by Vapnik and his co-workers in 1990s, have been developed quickly during the last decade [16], and successfully applied to biological data mining [17], drug discovery [18,19] etc. Denoting the training sample as *S *= {(**x**, **y**)} ⊆ {ℝ^*n *^× {-1, 1}}^ℓ^, SVM discriminant hyperplane can be written as

*y *= sgn(⟨**w**·**x**⟩ + *b*)

where **w **is a weight vector, *b *is a bias. According to the generalization bound in statistical learning theory [20], we need to minimize the following objective function for a 2-norm soft margin version of SVM:

(7) $$\begin{array}{ll} {\text{minimize}}_{w,b} & \langle w\cdot w\rangle +C\Sigma_{i=1}^{\ell }\xi_{i}^{2}\\ \text{subject to} & y_{i}(\langle w\cdot x_{i}\rangle +b)\geq 1-\xi_{i},i=1,...,\ell , \end{array}$$

in which, slack variable *ξ*_*i *_is introduced when the problem is infeasible. The constant *C *> 0 is a penalty parameter, a larger *C *corresponds to assigning a larger penalty to errors.

### Data Sets

Two microarray data sets used in our study are listed in Table 6. They are briefly described as below, and the corresponding C4.5 format versions are available at [21]. We do not use the original split by their authors, we merge the data set before using it.

**Table 6:** Experimental data sets

Data sets	Number of examples	Class ratio	Number of genes
Colon	62	22/40	2,000
Leukemia	72	25/47	7,129

**Colon **used Affymetrix oligonucleotide arrays to monitor expressions of over 6,500 human genes with samples of 40 tumor and 22 normal colon tissues. Expression of the 2,000 genes with the highest minimal intensity across the 62 tissues were used in the analysis [2].

**Leukemia **The acute leukemia data set was published by [1], which consists of 72 bone marrow samples with 47 ALL and 25 AML. The gene expression intensities are obtained from Affymetrix high-density oligonucleotide microarrays containing probes for 7,129 genes.

### Experimental settings

We use the stratified 10-fold cross-validation procedure, where each data set is firstly merged and then split into ten subsets of equal size. Each subset is used as a test set once, and the corresponding left subsets are combined together and used as the training set. Within each cross-validation fold, the gene expression data is standardized. The expressions of the training set are transformed to zero mean and unit standard deviation across samples, and the test set are transformed according to the means and standard deviations of the corresponding training set. We use 10 fold cross validation because the 10 × 10 cross-validation measurement is more reliable than the randomized re-sampling test strategy and the leave-one-out cross-validation due to the correlations between the test and training sets, some detail discussions can be found at [22].

**Figure 7:**
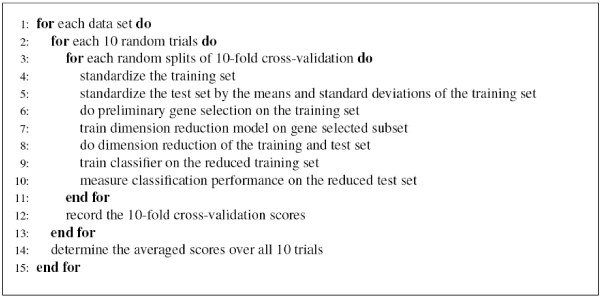
Experimental procedure for comparing different algorithms.

The linear Support Vector Machine (SVM) with *C *= 1 is used as the classifier, which is trained on the training set to predict the label of test samples. Figure 7 contains pseudo-code to describe the complete 10 × 10 cross-validation measurement procedure.

In order to precisely characterize the performance of different learning methods, we define several performance measures below (see [23]). Here TP, TN, FP, and FN, stand for the number of true positive, true negative, false positive, and false negative samples, respectively.

Sensitivity is defined as $\frac{TP}{TP+FN}$ and is also known as Recall.

Specificity is defined as $\frac{TN}{TN+FP}$.

BACC (Balanced Accuracy) is defined as $\frac{1}{2}\left(\frac{TP}{TP+FN}+\frac{TN}{TN+FP}\right)$, which defines the average of sensitivity and specificity.

Precision is defined as $\frac{TP}{TP+FP}$.

PPV (Positive Predictive Value) is defined as $\frac{TP}{TP+FP}$.

NPV (Negative Predictive Value) is defined as $\frac{TN}{TN+FN}$.

Correction is defined as $\frac{TP+TN}{TP+TN+FP+FN}$ and measures the overall percentage of samples correctly classified.

## Competing interests

The authors declare that they have no competing interests.

## Authors' contributions

Guo-Zheng Li and Xue-Qiang Zeng proposed the idea, designed the experiments and wrote the paper; Xue-Qiang Zeng performed experiments; Geng-Feng Wu helped in writing the paper; Mary Qu Yang helped design the experiments; Jack Y. Yang conceived and guided the project.

## References

[B1] Golub TR, Slonim DK, Tamayo P, Huard C, Gaasenbeek M, Mesirov JP, Coller H, Loh ML, Downing JR, Caligiuri MA, Bloomfield CD, Lander ES (1999). Molecular Classification of Cancer: Class Discovery and Class Prediction by Gene Expression. Bioinformatics & Computational Biology.

[B2] Alon U, Barkai N, Notterman DA, Gish K, Ybarra S, Mack D, Levine AJ (1999). Broad patterns of gene expression revealed by clustering analysis of tumor and normal colon tissues probed by oligonucleotide arrays. Proceedings of the National Academy of Sciences of the United States of America.

[B3] Antoniadis A, Lambert-Lacroix S, Leblanc F (2003). Effective dimension reduction methods for tumor classification using gene expression data. Bioinformatics.

[B4] Nguyen DV, David DM, Rocke M (2004). On partial least squares dimension reduction for microarray-based classification: a simulation study. Computational Statistics & Data Analysis.

[B5] Dai JJ, Lieu L, Rocke D (2006). Dimension reduction for classification with gene expression data. Statistical Applications in Genetics and Molecular Biology.

[B6] Yu L, Liu H (2004). Redundancy Based Feature Selection for Microarray Data. Proc 10th ACM SIGKDD Conf Knowledge Discovery and Data Mining.

[B7] Yu L, Liu H (2004). Efficient Feature Selection Via Analysis of Relevance and Redundancy. Journal of Machine Learning Research.

[B8] Guyon I, Elisseefi A (2003). An Introduction to Variable and Feature Selection. Journal of Machine Learning Research.

[B9] Forman G (2003). An Extensive Empirical Study of Feature Selection Metrics for Text Classification. Journal of Machine Learning Research.

[B10] Hall MA, Holmes G (2003). Benchmarking attribute selection techniques for discrete class data mining. IEEE Transactions on Knowledge and Data Engineering.

[B11] Jolliffe IT (2002). Principal Component Analysis.

[B12] Wold S, Ruhe A, Wold H, Dunn W (1984). Collinearity problem in linear regression. The partial least squares (PLS) approach to generalized inverses. SIAM Journal of Scientific and Statistical Computations.

[B13] Boulesteix AL, Strimmer K (2006). Partial Least Squares: A Versatile Tool for the Analysis of High-Dimensional Genomic Data. Briefings in Bioinformatics.

[B14] Nguyen DV, Rocke DM (2002). Multi-class cancer classification via partial least squares with gene expression profiles. Bioinformatics.

[B15] Nguyen DV, Rocke DM (2002). Tumor classification by partial least squares using microarray gene expression data. Bioinformatics.

[B16] Cristianini N, Shawe-Taylor J (2000). An Introduction to Support Vector Machines.

[B17] Guyon I, Weston J, Barnhill S, Vapnik V (2002). Gene Selection for Cancer Classification Using Support Vector Machines. Machine Learning.

[B18] Xue Y, Li ZR, Yap CW, Sun LZ, Chen X, Chen YZ (2004). Effect of Molecular Descriptor Feature Selection in Support Vector Machine Classification of Pharmacokinetic and Toxicological Properties of Chemical Agents. Journal of Chemical Information & Computer Science.

[B19] Bhavani S, Nagargadde A, Thawani A, Sridhar V, Chandra N (2006). Substructure-Based Support Vector Machine Classifiers for Prediction of Adverse Effects in Diverse Classes of Drugs. Journal of Chemical Information and Modeling.

[B20] Vapnik V (1998). Statistical Learning Theory.

[B21] Li J, Liu H (2002). Kent Ridge Bio-medical Data Set Repository. http://www.cs.shu.edu.cn/gzli/data/mirror-kentridge.html.

[B22] Dietterich TG (1998). Approximate Statistical Tests for Comparing Supervised Classification Learning Algorithms. Neural Computation.

[B23] Levner I (2005). Feature Selection and Nearest Centroid Classification for Protein Mass Spectrometry. BMC Bioinformatics.

